# When Expertise Goes Undercover: Exploring the Impact of Perceived Overqualification on Knowledge Hiding and the Mediating Role of Future Work Self-Salience

**DOI:** 10.3390/bs15081134

**Published:** 2025-08-20

**Authors:** Xiaoyun Ren, Di Wu, Qian Zhang, Haitianyu Lin

**Affiliations:** 1 School of Economics and Management, Zhejiang Sci-Tech University, Hangzhou 310018, China; renxiaoyun@zstu.edu.cn (X.R.); zhangqianjessie@zstu.edu.cn (Q.Z.); 2 School of Management, Zhejiang Gongshang University Hangzhou College of Commerce, Hangzhou 311508, China; linhty@126.com

**Keywords:** perceived overqualification, knowledge hiding, person–environment fit, future work self-salience, growth mindset

## Abstract

Grounded in the person–environment fit theory and an identity-based perspective, this study investigated the relationship between perceived overqualification and knowledge hiding, focusing on the mediating role of future work self-salience and the moderating role of the growth mindset. We suggest that perceived overqualification as a person–job misfit would negatively impact employees’ salient hoped-for work identities, representing a low level of future work self-salience. The diminished salience of a future work self leads employees to hide their knowledge. Furthermore, the growth mindset exacerbates the negative impact of perceived overqualification. We conducted a three-wave survey with 482 employees from knowledge-intensive industries. The results revealed that perceived overqualification boosted knowledge hiding by decreasing employees’ future work self-salience. The growth mindset enhanced the negative relationship between perceived overqualification and future work self-salience. Thus, the indirect effect of perceived overqualification on knowledge hiding via future work self-salience was more significant for those with a stronger growth mindset. Our findings contribute to the literature on person–job fit and knowledge behavior while providing practical insights for managing and guiding talented employees in knowledge management.

## 1. Introduction

Overqualification refers to an employment situation where individuals possess more education, skills, and experiences than their job requires (Maynard & Parfyonova, 2013; Erdogan & Bauer, 2021). With increasing competition in the labor market and economic fluctuations, overqualification has become prevalent among employees, raising significant concerns for both practitioners and scholars (Rose, 2017). Typically, overqualification can be assessed objectively by comparing an individual’s education and experience levels to job demands, or subjectively through the individual’s perception of having excessive qualifications for their role (Liu et al., 2015). The subjective assessment, known as perceived overqualification, is a more proximal predictor of work attitudes and behaviors (Maynard & Parfyonova, 2013; Khan et al., 2023). Anecdotal evidence shows that perceived overqualification is closely associated with negative work outcomes, including job dissatisfaction, lower organizational commitment, and undesirable performance (Harari et al., 2017; Erdogan & Bauer, 2021).

Nevertheless, one of the purported benefits managers may anticipate from overqualified employees is their potential to promote organizational knowledge management. For example, organizations or managers could encourage overqualified employees to share valuable information and mentor less-experienced colleagues (Khan et al., 2023). Contrary to this expectation, researchers suggest that employees may be reluctant to disclose their knowledge to others, as knowledge is a powerful resource that helps employees to obtain competitive advantages and maximize their interests (Shafique et al., 2023). Empirical studies also indicate that overqualified employees often hesitate to assist their peers and may deliberately conceal their expertise (Erdogan et al., 2020; Shafique et al., 2023). This poses a paradox: although employees with extensive qualifications are presumed to expand the organization’s knowledge base, they may withhold their intellect (Ma & Zhang, 2022). Knowledge hiding is a form of knowledge withholding, which captures the intentional concealment of information requested by others (Connelly et al., 2012). Compared to general withholding that may or may not be explicitly solicited, knowledge hiding is characterized by its deliberate nature and involves possible strategies of hiding (Connelly et al., 2019). Thus, the present study aims to explore how and why overqualified employees intentionally hide their knowledge when approached for help.

Person–environment fit theory is an important approach to theorizing perceived overqualification and its influence (Kristof-Brown & Guay, 2011; Qiu et al., 2025). There are various types of fit based on different aspects of compatibility between individuals and their environment. For instance, person–organization fit describes the degree to which an individual’s values or beliefs match an organization’s values and culture (Edwards, 2008). This study adopts the person–job fit framework because it captures the essential features of perceived overqualification (Liu et al., 2015; Luksyte et al., 2022). Specifically, person–job fit focuses on the relationship between individuals’ characteristics and their job (Kristof-Brown & Guay, 2011). It includes demands–abilities fit, which assesses if employees’ capacities meet the job requirements, and needs–supplies fit, which refers to whether the job fulfills employees’ needs, desires, or preferences (Kristof-Brown et al., 2005). By definition, perceived overqualification could be identified as a person–job misfit, wherein employees’ knowledge, skills, and experiences significantly exceed their job requirements (demands–abilities misfit) (Zhao & Ma, 2023). Moreover, overqualified employees are unable to make full use of their valuable skills, leading to a lack of fulfillment in their need for challenging and intrinsically motivating roles (needs–supplies misfit) (He & Li, 2024).

According to the theory of person–job fit and related research, employees who feel a mismatch with their jobs have more unfavorable experiences, resulting in negative attitudinal and behavioral reactions in the workplace (Kristof-Brown et al., 2005). To address the above paradox that overqualified employees may conceal their expertise, this study examines the impact of perceived overqualification on a specific counterproductive behavior: knowledge hiding (Connelly et al., 2012). Knowledge is a high-value intellectual asset of employees, which usually requires a significant amount of time and effort to acquire (Connelly et al., 2019). Studies indicate that knowledge hiding is more likely to occur when individuals are dissatisfied with their work and experience high levels of stress (Anand et al., 2022). In this study, overqualified employees may hide their knowledge, as they may perceive their work as a constraint and feel frustrated when the jobs are inconsistent with their abilities or needs.

Recent studies have attempted to demonstrate the influence of perceived overqualification on knowledge hiding (Anand et al., 2022). The mechanism underlying this relationship has mainly concentrated on employees’ hostile emotions and cognitions toward others or organizations (e.g., anger, envy, distrust, and contempt) (Li et al., 2021; Venz & Nesher Shoshan, 2022; Khan et al., 2023). Despite the insights gained from existing studies, a noticeable research gap remains regarding whether having surplus qualifications that do not align with the job can impede employees’ self-concept or identity at work, leading them to hide their knowledge (Liu et al., 2015; Wu et al., 2023; He & Li, 2024). In line with the person–environment fit theory, a good person–job fit enables individuals to develop a positive work identity, whereas those who experience a mismatch find it hard to construct a clear identity (Edwards, 2008; Liu et al., 2015). For instance, studies find that overqualified employees often question their value, feel a lack of purpose, and struggle to envision future career paths (Erdogan & Bauer, 2021). These experiences are closely associated with their work identities. From an identity-based perspective, work-related identities reflect “who you are” and “what you do” at work, which is a critical component of a dynamic self-concept that shapes one’s thoughts and behaviors (Ashforth, 2000).

This study further integrates an identity-based perspective by conceptualizing a work identity as the future work self: the self in the future that encapsulates individually significant hopes and aspirations in relation to work (Strauss et al., 2012). We specifically focus on the salience of the future work self, defined as the extent to which the future work self is clear and easy to imagine (Strauss et al., 2012). In contrast to the general concept of work identity (e.g., vocational identity, commitment, and organization-based self-esteem), future work self-salience is future-oriented and captures individuals’ identification with their imagined future work lives and who they will become (Strauss & Kelly, 2016; Voigt & Strauss, 2024). Individuals with a salient future work self tend to display more proactive behaviors, such as developing new skills and networking with colleagues. Conversely, those lacking a salient future work self are more passive and unwilling to invest their energy or resources at work (Strauss et al., 2012; Taber & Blankemeyer, 2015). Applying this notion to the present study, overqualified employees who perceive a significant misalignment with their jobs may experience confusion about what they can achieve or who they will become. This diminished salience of the future work self may further lead to disengagement from work and an increase in knowledge hiding.

Moreover, the person–environment fit theory suggests that the influence of person–job fit could be moderated by the importance of the characteristics on which fit is assessed for the individual (Edwards, 2008; Maynard & Parfyonova, 2013). Perceived overqualification might be most influential among employees who highly value personal growth and development. These employees should be more sensitive to the mismatch between their abilities and job demands, and experience stronger negative feelings when they are unable to learn or improve in their work (Erdogan et al., 2020). Such intensive demands–abilities and needs–supplies mismatches make it increasingly difficult for employees to construct a salient future work self, exacerbating the negative consequences of overqualification (Liu et al., 2015). Accordingly, this study incorporates the growth mindset as the boundary condition in our theoretical model, which is defined as one’s belief that human attributes (e.g., skills, abilities, and intelligence) are malleable and can be developed through practices and efforts (Dweck, 2006; Han & Stieha, 2020). Compared to achievement motivation, which emphasizes the need to strive for success (e.g., mastering tasks or exceeding others), the growth mindset offers a meaning system that guides how people interpret and react to the environment (Dweck, 2006). Individuals with a higher growth mindset prefer the challenging task, as they interpret it as a valuable opportunity to improve themselves (Dweck & Yeager, 2019). Nevertheless, they are reluctant to perform tasks that restrict their capacities because the task may not meet their growth needs or help them recognize their potential in the future. Therefore, when employees possess a high growth mindset, perceived overqualification not only limits the chance to display their talents but also narrows their vision for future development, ultimately increasing knowledge hiding.

Combining person–environment fit theory with an identity-based perspective, perceived overqualification as a person–job misfit may reduce employees’ future work self-salience, leading employees to engage in knowledge hiding. Additionally, the growth mindset may exacerbate the negative impact of perceived overqualification. Therefore, we conduct a time-lagged survey, and our study contributes to the existing literature in the following ways. First, prior research based on the person–environment fit theory has primarily focused on the detrimental influences of perceived overqualification on general work behaviors (Liu et al., 2015). This study particularly links perceived overqualification with knowledge hiding, applying the person–environment fit theory to knowledge management. Second, our study examines the mediating effect of future work self-salience, which provides a new perspective for identifying the association between perceived overqualification and knowledge hiding (Strauss et al., 2012). Third, our research explores the moderating role of growth mindset, which offers novel insights into the conditions under which and for whom the consequences of overqualification would manifest in different patterns (Han & Stieha, 2020). It also responds to the call for more research on overqualification by incorporating individual dispositions (M. J. Zhang et al., 2016).

## 2. Theory and Hypotheses Development

### 2.1. Perceived Overqualification and Knowledge Hiding

Knowledge hiding refers to an individual intentionally withholding information in response to inquiries from others (Connelly et al., 2012). This behavior represents a deliberate concealment rather than the opposite of knowledge sharing that might be due to the absence of information (Connelly et al., 2012). Specifically, knowledge hiding is a multidimensional construct, which composed of three facets: playing dumb (i.e., individuals pretend not to know the knowledge that is requested), evasive hiding (i.e., individuals offer inappropriate or incomplete information), and rationalized hiding (i.e., the hider find excuses or reasons for not providing the knowledge to the requestor) (Connelly et al., 2019). According to Connelly et al. (2012), three dimensions could be measured individually or in combination, depending on the research question. This study assesses three dimensions because the overall construct of knowledge hiding is of interest.

Numerous studies have demonstrated the deleterious effects of knowledge hiding on employees’ performance (El-Kassar et al., 2022), interpersonal relations (Venz & Nesher Shoshan, 2022), and team effectiveness (Bogilović et al., 2017). Scholars and practitioners are keen to understand the causes of knowledge hiding in order to prevent and manage this issue (Anand et al., 2022). To date, the antecedents of knowledge hiding involve individual attributes (e.g., narcissism, machiavellianism; Pan et al., 2018), leader behavior (e.g., abusive supervision; Offergelt & Venz, 2023), and organizational culture (e.g., injustice; Jahanzeb et al., 2020). In general, negative work experiences are important triggers for knowledge hiding. For instance, researchers demonstrate that perceived abusive and unjust treatment leads employees to hide their knowledge (Connelly et al., 2019; Offergelt & Venz, 2023). Perceived overqualification is an unfavorable situation where employees have surplus qualifications that are not necessarily required by the job. Drawing on the person–job fit framework, people have innate needs that align with their jobs (Kristof-Brown & Guay, 2011). However, a poor person–job fit presents a stressful work state (Kristof-Brown et al., 2005). Employees with such negative experiences are more inclined to be absent or withdraw from work; they will be less productive and exhibit undesirable interpersonal behaviors, such as refusing to assist coworkers or even bullying others (Vandevelde et al., 2020; Vila-Vázquez et al., 2023).

We suggest that perceived overqualification, as a form of person–job misfit, may increase employees’ knowledge hiding. From the cognitive perspective, overqualified employees often feel that organizations do not respect their talents as they cannot fully utilize their abilities (Erdogan & Bauer, 2021; Ma & Zhang, 2022). Additionally, individuals tend to calculate the weight of their inputs and gains and compare the results with their peers (Khan et al., 2023). When overqualified employees feel that the inputs are disproportionate to the rewards received in relation to those whose qualifications match job demands (Cheng et al., 2020), they may perceive themselves as being treated unfairly and envy their colleagues (Li et al., 2021). Therefore, employees with negative attitudes towards organizations or coworkers may be less concerned about collective interest and development, resulting in knowledge hiding. In terms of the emotional aspect, studies have found that overqualified employees report more negative emotions, such as anxiety, anger, and depression (Erdogan & Bauer, 2021; Ma & Zhang, 2022). They may tend to express frustration through passive and counterproductive behavior, such as concealing their knowledge from others (Li et al., 2021). For instance, Ma and Zhang (2022) found that overqualified employees experience a strong negative emotional state and hence show more knowledge hiding. Therefore, we propose the following hypothesis:

**H1:**  *Perceived overqualification is positively related to knowledge hiding.*

### 2.2. The Mediating Effect of Future Work Self-Salience

In line with the person–environment fit theory, a desirable person–job fit could foster and maintain positive work identities, whereas a poor person–job fit is more likely to provoke employees’ uncertainty regarding their career trajectory, leading to negative work behaviors (Kristof-Brown et al., 2005; Sales et al., 2023). We integrate the person–job framework with an identity-based perspective, suggesting that overqualified employees may hardly envision a clear career path or ideal self-representation in future work, and this diminished salience of their future work self leads to increased knowledge hiding.

Building upon the concept of a possible self, a future work self serves as a source of identity-based motivation that guides one’s behavior, which refers to the image of oneself in the future that embodies hopes and aspirations related to work (Markus & Nurius, 1986; Strauss et al., 2012). The motivational impact of future work selves is primarily determined by the degree to which they are clear and easily imaginable, known as future work self-salience (Strauss et al., 2012). Studies suggest that people envision their future work self based on their work experiences (Guan et al., 2017; Guo et al., 2022). For example, performing challenging tasks can help employees identify their strengths and weaknesses, enhancing their future work self-salience (Pu et al., 2022). Research demonstrates that employees can create a salient future work self when they realize their current knowledge or skills fall short of their job demands (Pu et al., 2022). The gap between their qualifications and the job demands provides a direction for their future career development. Additionally, employees with positive self-perceptions at work, such as a sense of meaning and self-esteem, would view themselves as capable and significant within their organizations (Cai et al., 2015). They are motivated to develop a salient future work self and behave proactively to achieve it. In contrast, employees who lack opportunities for exploration or have negative self-perceptions toward current work roles are less likely to form a clear and salient work self in the future (Strauss & Kelly, 2016; Guan et al., 2017).

We argue that perceived overqualification may decrease future work self-salience. Employees who perceive their qualifications as substantially exceeding the job requirements may have limited chances to approach challenging tasks and develop their potential at work (Erdogan & Bauer, 2021; Kim et al., 2021). This lack of skill utilization and improvement opportunities may impede employees from envisioning a clear work identity in the future. Meanwhile, overqualified employees also feel frustrated when they are unable to exercise their competencies or prove their worth (Harari et al., 2017), which results in negative self-perceptions, such as job meaninglessness, insecurity, and lower self-esteem (Liu et al., 2015; Zhao & Ma, 2023). Thus, unchallenging tasks and negative self-perceptions would prevent overqualified employees from constructing salient future work selves. On this basis, we propose the following hypothesis:

**H2:**  *Perceived overqualification is negatively related to future work self-salience.*

According to an identity-based perspective, employees who possess a low level of future work self-salience are reluctant to invest their energy and resources in the workplace (Strauss & Kelly, 2016; Voigt & Strauss, 2024). We further suggest that employees with lower future work self-salience are inclined to hide their knowledge.

On the one hand, employees with lower future work self-salience tend to avoid interpersonal interactions. For instance, a low level of future work self-salience usually correlates with poor performance in socializing and networking with coworkers (Y. Zhang et al., 2014; Taber & Blankemeyer, 2015). Researchers indicate that employees with lower future work self-salience are unclear about their career goals or directions, undermining their expectations of obtaining resources from others and their intentions to share resources (Strauss & Kelly, 2016). It manifests as less initiative or avoidance in interpersonal interactions, such as hiding their knowledge when approached with inquiries from colleagues.

On the other hand, employees who have lower future work self-salience may choose to hide their knowledge as a self-protection strategy. Studies indicate that these employees often feel uncertain and insecure about their career (Connelly et al., 2012; Connelly et al., 2019). In this case, demonstrating their competencies or disclosing themselves to others could be a risky endeavor, as they lack control over their future selves (Serenko & Bontis, 2016; Venz & Nesher Shoshan, 2022). Hence, we propose the following hypothesis:

**H3:**  *Future work self-salience is negatively related to knowledge hiding.*

Thus, it is reasonable to assume that perceived overqualification increases knowledge hiding by decreasing future work self-salience. We propose the following hypothesis:

**H4:**  *Future work self-salience mediates the relationship between perceived overqualification and knowledge hiding.*

### 2.3. The Moderating Effect of Growth Mindset

While overqualified employees may have a low level of future work self-salience and are not willing to disclose their knowledge, this effect may vary to the extent to which people notice and react to the mismatch (Kristof-Brown & Guay, 2011). Given that perceived overqualification represents poor feelings of person–job fit, employees prioritizing career development are particularly vulnerable in these situations (Liu et al., 2015). Therefore, perceived overqualification would be most influential for employees who are eager to learn and grow at work. They may experience greater uncertainty about their future work self and are at high risk of engaging in knowledge hiding.

A growth mindset is a lay belief about the plasticity of human attributes, which shapes how individuals interpret and respond to the environment (Dweck, 2006). People with a high growth mindset view attributes as malleable and can be developed through practice and effort. Thus, they are more inclined to take on challenges and expand their capacities (Dweck & Yeager, 2019). Those who endorse a low growth mindset (a fixed mindset) believe their qualities are relatively stable. They tend to avoid situations that exceed their abilities and prefer tasks with predictable outcomes (Dweck, 2006). The growth mindset has been utilized to help individuals, especially underperforming ones, overcome difficulties and attain self-improvement (Dweck & Yeager, 2019). Nevertheless, when individuals have mastered the tasks, the effectiveness of the growth mindset is less pronounced as there is limited room for improvement (Walton & Wilson, 2018; Yeager & Dweck, 2020). Moreover, the growth mindset may yield adverse effects in the context of task underload. For instance, overqualified employees who value their competencies and growth display more counterproductive behavior and higher turnover intentions (Cheng et al., 2020). Researchers suggest that these employees are more likely to experience unfavorable self-perceptions, such as a lack of meaning and low self-efficacy, which results in more negative consequences at work (Maynard & Parfyonova, 2013; M. J. Zhang et al., 2016).

In the present study, we argue that a growth mindset may aggravate the negative impact of perceived overqualification on future work self-salience. Individuals with a growth mindset would more proactively envision and pursue their future development because they believe their characteristics can be developed (Dweck, 2006). This sense of potential progress motivates individuals to devote themselves to the work and consider challenging tasks as an opportunity to achieve a better self-representation. However, perceived overqualification may restrict such attempts (Cheng et al., 2020). When employees have growth needs that are not satisfied, they are more likely to interpret the overqualification as an obstacle to imagining a clear future work self. Nevertheless, employees with a lower growth mindset may not expect to learn on the job because they believe that human attributes are largely fixed (M. J. Zhang et al., 2016; Dweck & Yeager, 2019). Thus, they may be less sensitive to overqualification and would not necessarily experience a decrease in future work self-salience. Given that the growth mindset influences how individuals interpret and react to the environment based on whether they can improve themselves in the future, we focus on its moderating effect on the relationship between perceived overqualification and future work self-salience. Thus, we propose the following hypothesis:

**H5:**  *Growth mindset moderates the relationship between perceived overqualification and future work self-salience, such that the relationship is stronger for employees with a high level of growth mindset.*

**Figure 1:**
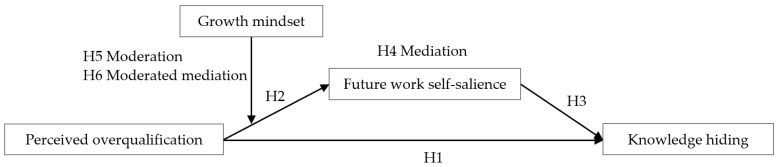
Hypothesized Model.

Taken together, it suggests a moderated mediation model (Figure 1) in which future work self-salience mediates the influence of perceived overqualification on knowledge hiding, and the growth mindset moderates the relationship between perceived overqualification and future work self-salience. As a consequence, the indirect effect of perceived overqualification on knowledge hiding via future work self-salience would be more substantial at a high level of the growth mindset.

**H6:**  *Growth mindset moderates the indirect relationship between perceived overqualification and knowledge hiding through future work self-salience, such that the indirect effect is enhanced for employees with a high level of the growth mindset.*

## 3. Materials and Methods

### 3.1. Sample and Procedure

We gathered data from knowledge-intensive industries in China, including information and communication technology, biomedical R&D, and automobile R&D companies. Consistent with the research on perceived overqualification and knowledge behavior, the selected industries are mainly knowledge-oriented and innovation-based (Ma & Zhang, 2022; Khan et al., 2023). Participants from the information and communication technology company are involved in designing and developing hardware devices and software applications. They provide global operators with network planning, construction, optimization, and operations services. In the biomedical R&D company, participants are engaged in drug research and development. Meanwhile, in the automobile R&D company, participants focus on designing automobile parts, developing power systems, and researching intelligent driving. Previous research has shown that knowledge-intensive industries place a great value on employees’ expertise (Wu et al., 2023). Employees in these industries typically possess high levels of knowledge, skills, and abilities. The perception of overqualification might be more prominent among such employees. Additionally, innovative work requires effective team communication and collaboration. Thus, interpersonal knowledge behavior might frequently occur in these selected industries (Ma & Zhang, 2022).

We sought permission from the companies’ management teams. With the help of the human resource department, we obtained lists of employees whose work requires specific knowledge. Two of the researchers visited their work sites and explained the research purpose. In the end, 785 employees volunteered to participate. All participants were guaranteed anonymity and assured that their responses would remain confidential. The investigation adopted a three-wave design to reduce the common method bias. Before the surveys, two researchers and six assistants assigned a unique ID to each participant and pre-coded the questionnaires to ensure accurate matching for each survey. Researchers distributed the questionnaires to participants, asked them to complete the surveys, and returned them directly to research assistants. Then, another two researchers and two assistants collated the anonymous questionnaires with IDs and input the information.

We collected data in July 2023 (Time 1), August 2023 (Time 2), and September 2023 (Time 3). At Time 1, employees provided demographic information and evaluated their perceived overqualification and growth mindset. One month later, at Time 2, employees reported their future work self-salience. Another month later, at Time 3, employees completed the ratings of knowledge hiding. We distributed questionnaires to 785 employees at Time 1. A total of 746 valid questionnaires were returned, yielding a 95.03% response rate. There was some attrition from Time 1 to Time 2, with 594 questionnaires received, resulting in a participation rate of 75.67%. By Time 3, we received 488 questionnaires, with a 62.17% response rate in this phase. After excluding six questionnaires with more than half of the items unanswered, our final sample consisted of 482 employees. Our response rate was similar to the previous research on perceived overqualification using time-lagged methods (Li et al., 2021; Khan et al., 2023; Shafique et al., 2023). Given that perceived overqualification was collected at Time 1 with a high response rate, we further examined the potential influence of non-response bias in this study. The results found that there was no significant difference in the perceived overqualification scores between our final sample (*M* = 3.14, *SD* = 0.89) and the missing sample (*M* = 3.20, *SD* = 0.94) (*t* = 0.87, *p* = 0.39).

Among 482 employees, 37.10% worked in the information and communication technology industry, 35.90% in the biomedical industry, and 27% in the automobile industry. The ages of participants ranged from 22 to 54 (*M* = 33.72, *SD* = 5.66), and their tenure in the current organization ranged from 1 to 27 (*M* = 8.24, *SD* = 4.64). In total, 51.90% of participants were male, and 86.80% held bachelor’s degrees or above.

### 3.2. Measures

Surveys were administered in China. All questionnaires were originally in English. We followed a two-way translation procedure to develop the Chinese version of the surveys. The measurement items can be found in Appendix A, Table A1. We used a five-point Likert-type scale for all hypothesized variables, ranging from 1 (strongly disagree) to 5 (strongly agree).

#### 3.2.1. Perceived Overqualification (Time 1)

We used the four-item scale by Johnson and Johnson (1997) to assess perceived overqualification. A sample item was “My work experience is more than necessary to do my present job.” The Cronbach’s alpha for this scale was 0.85.

#### 3.2.2. Growth Mindset (Time 1)

The eight-item scale adapted from Dweck et al. (1995) was utilized to measure employees’ growth mindset. A sample item was “Everyone, no matter who they are, can significantly change their basic characteristics.” The Cronbach’s alpha for this scale was 0.92.

#### 3.2.3. Future Work Self-Salience (Time 2)

Strauss et al. (2012) developed a five-item scale to measure employees’ future work self-salience. A sample item was “I am very clear about who and what I want to become in my future work.” The Cronbach’s alpha for this scale was 0.87.

#### 3.2.4. Knowledge Hiding (Time 3)

Knowledge hiding was measured by a twelve-item scale developed by Connelly et al. (2012), which involved playing dumb, evasive hiding, and rationalized hiding. Sample items were “Said that I did not know, even though I did,” “Agreed to help him/her but never really intended to,” and “Explained that I would like to tell him/her, but was not supposed to.” The Cronbach’s alpha for the scale was 0.93.

#### 3.2.5. Control Variables

This study controlled the potential influences of employees’ demographic attributes on knowledge hiding, including age and gender. We also controlled employees’ education and tenure, as the two factors can indicate work-related knowledge to a certain extent, which might influence knowledge hiding (Erdogan & Bauer, 2021; Ma & Zhang, 2022). Gender was dummy coded, “1” for male and “2” for female. Educational background was categorized into four levels. “1” for senior high school or below, “2” for junior college degree, “3” for bachelor’s degree, and “4” for master’s degree or higher. Age and tenure were measured in years as continuous variables.

### 3.3. Analytic Strategy

All variables were conceptualized and measured at the individual level. First, we used Mplus 8.0 to perform a confirmatory factor analysis to ensure the discriminant validity of the constructs (perceived overqualification, future work self-salience, knowledge hiding, growth mindset) and reported the correlations. Next, we adopted SPSS 26 to examine the relationship among hypothetical variables. We performed multiple linear regressions while controlling for the demographic variables, including regression models of knowledge hiding and regression models of future work self-salience. Then, we used bootstrapping analyses (with a specified bootstrap sample of 5000) to test the mediating and moderating models.

## 4. Results

### 4.1. Common Method Bias

As recommended by Podsakoff et al. (2003), this study implemented ex-ante and ex-post approaches to reduce the common method bias. We adopted a three-wave data collection method and set several reverse items in the questionnaires before launching the surveys. In the post-measures, we applied the Harman test. The results showed that the first principal component explained 38.80% of the variance, less than the suggested threshold of 50% (Khan et al., 2023). In addition, we combined all hypothesized variables into one factor; the single-factor fitting index was not optimal (*χ*^2^/*df* = 10.55, TLI = 0.42, CFI = 0.46, RMSEA = 0.14, SRMR = 0.18). The results indicated no significant influence of common method variance on the research outcomes (Podsakoff et al., 2003).

### 4.2. Confirmatory Factor Analysis

We conducted confirmatory factor analyses using maximum-likelihood estimation, which consisted of four factors: perceived overqualification, future work self-salience, knowledge hiding, and growth mindset. Table 1 presents the discriminant validity tests for the four models. Compared with the alternative models, the four-factor model yielded a better fit than any other model (*χ*^2^/*df* = 1.06, TLI = 0.99, CFI = 0.99, RMSEA = 0.01, SRMR = 0.03). The results suggested sufficient distinctiveness among the four constructs in the hypothesized model.

We tested the convergent validity of hypothesized variables. Previous research suggested that convergent validity occurs when the item loading > 0.50, AVE > 0.50, and CR > 0.70 (Hair et al., 2015). As presented in Table 2, among the four constructs, the values of item loadings were greater than 0.70, AVEs were greater than 0.50, and CRs were greater than 0.90. Our hypothesized model works well for measuring the four constructs.

### 4.3. Descriptive Statistics

Table 3 shows the means, standard deviations, and correlations for all measured variables. Perceived overqualification was positively related to knowledge hiding (*r* = 0.29, *p* < 0.001) and negatively related to future work self-salience (*r* = −0.26, *p* < 0.001). Future work self-salience was negatively related to knowledge hiding (*r* = −0.28, *p* < 0.001). The correlation results provided initial support for the theoretical hypotheses.

### 4.4. Hypothesis Testing

Table 4 shows the results of multiple linear regressions. We regressed control variables, followed by perceived overqualification, future work self-salience, and growth mindset on knowledge hiding (Model 1–2). Then, we sequentially regressed control variables, perceived overqualification, growth mindset, and their interaction on future work self-salience (Model 3–5). The results indicated that perceived overqualification positively influenced knowledge hiding (*B* = 0.14, *p* < 0.001, 95%CI [0.09, 0.20], Model 2), which supported H1.

Consistent with the mediation hypothesis, perceived overqualification negatively predicted FWSS (*B* = −0.23, *p* < 0.001, 95%CI [−0.31, −0.16], Model 4). FWSS negatively predicted knowledge hiding (*B* = −0.15, *p* < 0.001, 95%CI [−0.22, −0.10], Model 2). H2 and H3 were confirmed. A bootstrapping analysis found that future work self-salience partially mediated the relationship between perceived overqualification and knowledge hiding (Indirect effect = 0.04, 95%CI [0.02, 0.06]). H4 was supported.

**Figure 2:**
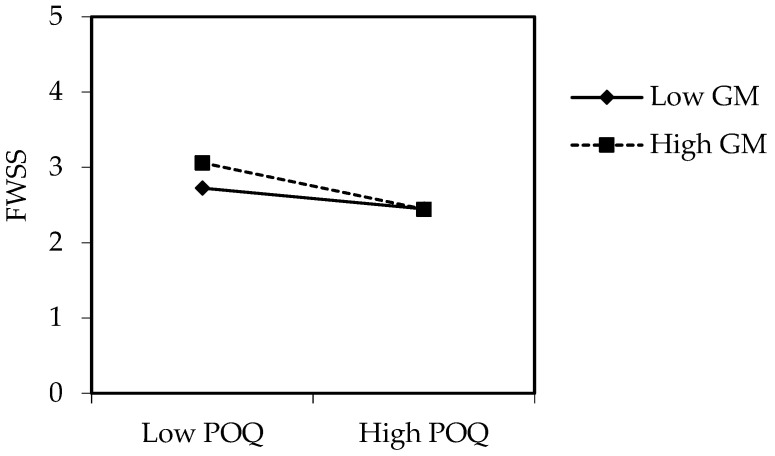
The interaction of POQ and GM on FWSS. Note: *N* = 482; POQ = perceived overqualification; GM = growth mindset; FWSS = future work self-salience. Low and high conditions represent −1SD and +1 SD around the mean of the variables.

Furthermore, the regression results demonstrated an interactive effect of perceived overqualification and growth mindset on future work self-salience (*B* = −0.11, *p* = 0.007, 95%CI [−0.19, −0.03], Model 5). We plotted the interaction using simple slopes at high (1 SD above the mean) and low (1 SD below the mean) levels of growth mindset to test H5. As depicted in Figure 2, growth mindset moderated the relationship between perceived overqualification and future work self-salience (*F* (1,474) = 4.57, *p* = 0.03). Perceived overqualification has a stronger negative impact on future work self-salience for employees with a high level of growth mindset (*B* = −0.35, 95%CI [−0.50, −0.20]) than for employees with a low level of growth mindset (*B* = −0.16, 95%CI [−0.25, −0.06]). H5 was supported.

Finally, we conducted the moderated mediation test (Table 5), and the results found that growth mindset moderated the mediating effect of future work self-salience on the relationship between perceived overqualification and knowledge hiding (Index of moderated mediation = 0.02, 95%CI [0.003, 0.04]). The indirect effect of perceived overqualification on knowledge hiding via future work self-salience was more significant for employees with a high level of growth mindset (Conditional indirect effect = 0.05, 95%CI [0.03, 0.09]) than for those with a low level of growth mindset (Conditional indirect effect = 0.02, 95%CI [0.01, 0.04]), which confirmed H6.

## 5. Discussion

### 5.1. Theoretical Implications

The findings of this study have three noteworthy theoretical implications. Firstly, we conceptualize perceived overqualification as a form of person–job misfit and demonstrate its impact on knowledge hiding. This result aligns with the person–environment fit theory, confirming the detrimental consequences of a person–job misfit (Kristof-Brown et al., 2005). Previous research on the person–job misfit typically focuses on situations where employees’ qualities do not meet general work standards (Kristof-Brown & Guay, 2011). Edwards (2008) suggests that a person–job misfit occurs when job demands exceed one’s capabilities, as well as when environmental demands fall short of one’s capabilities. This study explores the form of the misfit when employees possess more qualifications than are required for their jobs (Maynard & Parfyonova, 2013). We find that this type of person–job misfit, however, increases employees’ knowledge hiding. Our study responds to the call for conceptualizing perceived overqualification through the lens of person–environment fit theory (Liu et al., 2015; Li et al., 2021) and extends its negative influence to knowledge behavior. Moreover, this finding is consistent with the research on knowledge hiding. Studies have proven that undesirable traits or work conditions are critical antecedents for knowledge hiding (Anand et al., 2022). Our research identifies person–job misfit as a key factor that could cause knowledge hiding, enriching the knowledge management literature.

Second, this study reveals the mediating role of future work self-salience on the relationship between perceived overqualification and knowledge hiding. Overqualified employees struggle to develop a clear work self-concept for their future, as their jobs are inconsistent with their abilities or needs. The lack of future work self-salience boosts their knowledge hiding. The finding is in accordance with the person–environment fit theory, which indicates that person–job misfit prevents individuals from forming and maintaining positive work identities, thereby increasing their counterproductive behavior (Kristof-Brown & Guay, 2011; Strauss & Kelly, 2016). More importantly, we expand the scope of the existing research by adopting an identity-based perspective. Previous studies have mainly interpreted knowledge hiding as an aggressive reaction to perceived overqualification in terms of equity, relative deprivation, and social comparison theories (Anand et al., 2022; Khan et al., 2023). While knowledge hiding is regarded as a counterproductive behavior, individuals who engage in such actions may not always anticipate harming others or retaliation (Pereira & Mohiya, 2021). For instance, employees may experience a threat to their work identity when they perceive themselves as overqualified for the job. Consequently, they would withhold their knowledge—not necessarily out of a desire to undermine others or the organization, but due to their uncertainties about their future work (Strauss et al., 2012). Our study reveals that knowledge hiding among overqualified employees could be attributed to an unfavorable self-concept regarding their future work.

This study further demonstrates that the growth mindset amplifies the negative impact of perceived overqualification. The finding is consistent with the person–environment fit theory, which posits that fit-outcome relationships can be moderated by individuals’ traits or values (Kristof-Brown et al., 2005; Maynard & Parfyonova, 2013). Previous research indicates that employees who value their competencies and learning are more likely to be troubled by and react strongly to perceived overqualification (M. J. Zhang et al., 2016; Cheng et al., 2020). This study focuses on the growth mindset, which represents the belief that encourages individuals to take on challenges and pursue more learning goals that expand their abilities (Dweck & Yeager, 2019). We find that overqualified employees with a strong growth mindset struggle more to envision a promising future in their work, leading to lower future work self-salience and hence promoting their knowledge hiding. While prior research has highlighted the positive influences of a growth mindset (e.g., improving one’s abilities and resilience in adversity) (Heslin et al., 2020; Berg et al., 2023), this study reveals a significant aspect of its potential downsides. When individuals perceive themselves as overqualified for their assigned tasks, they not only miss the opportunity to acquire new skills but also fail to fully utilize their abilities (Yeager & Dweck, 2020). Such a misfit causes a lower future work self, resulting in more negative behavior, such as knowledge hiding. Therefore, our finding also contributes to the literature on growth mindset by revealing its potential negative effects for overqualified employees.

### 5.2. Practical Implications

This study provides insights for business practitioners as well. Due to intense competition in the job market, an increasing number of candidates hold positions below their actual qualifications and capabilities (Rose, 2017; Khan et al., 2023). Our study suggests that perceived overqualification could lead to undesirable work behaviors. Thus, organizations and human resource departments should prioritize the recruitment of candidates whose qualifications closely align with the demands of the job (Kristof-Brown & Guay, 2011), which enhances the utilization of their competencies in future endeavors.

Given that overqualified employees engage in knowledge hiding because of the lack of hope and aspirations toward their future work, organizations could implement career counseling programs that offer future-oriented guidance (Strauss et al., 2012). For instance, it is recommended for counselors to develop team activities such as brainstorming and focused interviews that encourage employees to identify elements of their work self-concept in the future (Lin et al., 2024). Mental time travel is also an ideal activity for visualizing vivid images, enabling employees to envision their future and approach their potential work role (Guo et al., 2022). Furthermore, scholars have stressed the importance of leaders in cultivating employees’ salient future work selves (Strauss et al., 2012). Organizations should encourage leadership practices that emphasize coaching and communicating visions. Leaders can provide detailed feedback and articulate the organization’s future narratives and collective objectives to subordinates. It can help employees acknowledge their strengths and weaknesses and connect their future work self-concept with the broader organizational vision (Guo et al., 2022).

Finally, this study indicates that growth mindset interventions should take into account employees’ current work situations, for instance, whether they are overqualified in their jobs. Otherwise, the interventions might have the opposite effect (M. J. Zhang et al., 2016). Especially in high-tech industries such as information and communication technology and R&D companies, organizations highly encourage their employees to have a growth mindset and implement related interventions, as it may help employees develop new products and make a technical breakthrough (Han & Stieha, 2020; Heslin et al., 2020). However, this study finds that when overqualified employees possess a high growth mindset, they perceive a stronger person–job misfit, which hinders their work self-concept and gives rise to counterproductive behavior. Therefore, managers could comprehensively assess the possibility of an individual’s growth and progress in the current working environment before launching the growth mindset interventions.

### 5.3. Limitations and Future Directions

Our study has some limitations that could be addressed in future research. Although we collected variables in three separate sessions to reduce the common method bias, the results were based on cross-sectional data. Future studies could adopt a longitudinal method to provide causal evidence for our hypothesized model. Alternatively, researchers could manipulate employees’ perceptions of overqualification and then measure their knowledge hiding in a scenario-based experiment.

Furthermore, all the variables in our model were assessed at the employee level to better capture how and when employees’ work self-concept influences their responses to perceived overqualification. However, we have not involved factors from organizational or leader levels that may moderate the relationships between perceived overqualification, future work self-salience, and knowledge hiding. As mentioned above, organizational support and effective leadership may buffer the negative consequences of perceived overqualification by promoting clear and accessible future work selves for employees (Guo et al., 2022). Future research could explore this issue further.

## 6. Conclusions

Grounded in person–environment fit theory and an identity-based perspective, this study examines the influence of perceived overqualification on knowledge hiding. The results demonstrate that perceived overqualification increases employees’ knowledge hiding by diminishing their future work self-salience. In addition, the growth mindset enhances the negative relationship between perceived overqualification and future work self-salience. Consequently, the indirect effect of perceived overqualification on knowledge hiding through future work self-salience is more substantial for employees with a strong growth mindset.

## Figures and Tables

**Table 1:** Confirmatory factor analysis.

Models	*χ*^2^/*df*	RMSEA	SRMR	CFI	TLI
POQ, FWSS, KH, GM	1.06	0.01	0.03	0.99	0.99
POQ, FWSS + KH, GM	3.44	0.07	0.08	0.86	0.85
POQ + FWSS + KH, GM	5.10	0.09	0.10	0.77	0.75
POQ + FWSS + KH + GM	10.55	0.14	0.18	0.46	0.42

Note: *N* = 482; POQ = perceived overqualification; FWSS = future work self-salience; KH = knowledge hiding; GM = growth mindset.

**Table 2:** Item loadings.

Construct	Estimate	SE	AVE	CR
POQ				
1	0.76	0.02	0.58	0.91
2	0.73	0.02		
3	0.78	0.03		
4	0.77	0.02		
FWSS				
1	0.72	0.02	0.57	0.92
2	0.76	0.02		
3	0.71	0.02		
4	0.77	0.02		
5	0.80	0.02		
KH				
1	0.70	0.03	0.53	0.96
2	0.73	0.03		
3	0.75	0.03		
4	0.73	0.02		
5	0.75	0.02		
6	0.76	0.02		
7	0.70	0.03		
8	0.73	0.03		
9	0.73	0.03		
10	0.75	0.02		
11	0.70	0.03		
12	0.69	0.03		
GM				
1	0.74	0.02	0.58	0.95
2	0.78	0.02		
3	0.78	0.02		
4	0.69	0.02		
5	0.76	0.02		
6	0.74	0.02		
7	0.80	0.02		
8	0.81	0.02		

Note: *N* = 482; POQ = perceived overqualification; FWSS = future work self-salience; KH = knowledge hiding; GM = growth mindset. AVE = average variance extracted; CR = Composite reliability.

**Table 3:** Descriptives and correlations between variables.

	*M*	*SD*	1	2	3	4	5	6	7	8
1 Gender	1.48	0.50								
2 Age	33.72	5.66	−0.07							
3 Education	3.18	0.65	0.004	0.01						
4 Tenure	8.24	4.64	−0.001	0.64 ***	−0.07					
5 POQ	3.14	0.89	0.02	−0.06	0.04	−0.06	(0.85)			
6 FWSS	2.90	0.77	−0.09	0.05	0.05	0.004	−0.26 ***	(0.87)		
7 KH	2.91	0.54	0.02	−0.001	0.04	−0.01	0.29 ***	−0.28 ***	(0.93)	
8 GM	3.24	0.85	0.02	−0.001	0.02	0.08	0.06	0.11 *	−0.01	(0.92)

Note: *N* = 482; Cronbach alpha reliabilities are reported in parentheses. Gender: 1 = male, 2 = female; Education: 1 = senior high school or below, 2 = junior college degree, 3 = bachelor’s degree, 4 = master’s degree or above. POQ = perceived overqualification; FWSS = future work self-salience; KH = knowledge hiding; GM = growth mindset. * *p* < 0.05, *** *p* < 0.001.

**Table 4:** Multiple linear regressions.

	KH		FWSS		
	Model 1	Model 2	Model 3	Model 4	Model 5
Control variables					
Gender	0.02(0.05)	0.001(0.05)	−0.13(0.07)	−0.12(0.07)	−0.11(0.07)
Age	0.001(0.01)	0.003(0.01)	0.01(0.01)	0.01(0.01)	0.01(0.01)
Education	0.03(0.04)	0.03(0.04)	0.06(0.05)	0.07(0.05)	0.07(0.05)
Tenure	−0.001(0.01)	−0.001(0.01)	−0.01(0.01)	−0.01(0.01)	−0.01(0.01)
Predictor variables					
POQ		0.14(0.03) ***		−0.23(0.04) ***	−0.11(0.13)
GM		−0.001(0.03)		0.12(0.04) **	0.45(0.13) **
FWSS		−0.15(0.03) ***			
Interaction					
POQ × GM					−0.11(0.04) **
R^2^	0.002	0.13	0.01	0.10	0.11
F	0.24	23.02 ***	1.46	21.90 ***	7.25 **

Note: *N* = 482; Model reflects unstandardized coefficients with standard errors. POQ = perceived overqualification; FWSS = future work self-salience; KH = knowledge hiding; GM = growth mindset. ** *p* < 0.01, *** *p* < 0.001.

**Table 5:** Mediation, moderation, and moderated mediation tests.

	**POQ→FWSS→KH**	
Mediation of FWSS	*Effect* (*SE*)	95%CI
Direct effect	0.14 (0.03)	[0.09, 0.20]
Indirect effect	0.04 (0.01)	[0.02, 0.06]
	**POQ→FWSS**	
Moderation of GM	*Effect* (*SE*)	95%CI
Low GM	−0.16 (0.05)	[−0.25, −0.06]
High GM	−0.35 (0.08)	[−0.50, −0.20]
	**POQ→FWSS→KH**	
Moderated mediation	*Effect* (*SE*)	95%CI
Low GM	0.02 (0.01)	[0.01, 0.04]
High GM	0.05 (0.02)	[0.03, 0.09]

Note: *N* = 482; Model reflects unstandardized coefficients with standard errors; CI = confidence interval. POQ = perceived overqualification; FWSS = future work self-salience; KH = knowledge hiding.

## Data Availability

The raw data supporting the conclusions of this article will be made available by the authors on request.

## Appendix A

**Table A1:** Measurement items.

**Perceived overqualification (Johnson & Johnson, 1997)**
1. My work experience is more than necessary to do my present job (我的工作经验对我现在的工作来说绰绰有余) 2. My talents are not fully utilized on my job (我现在的工作并不能充分发挥我所有的才能) 3. My formal education overqualifies me for my present job (我的教育水平高于目前工作的要求) 4. Based on my skills, I am overqualified for the job I hold (我所拥有的工作技能高于这份工作的要求)
**Future work self-salience (Strauss et al., 2012)**
1. I am very clear about who and what I want to become in my future work (我非常清楚自己在未来工作中想成为什么样的人) 2. This future is very easy for me to imagine (这一未来对我来说非常容易想象) 3. What type of future I want in relation to my work is very clear in my mind （我对自己在工作方面想要的未来非常清晰） 4. I can easily imagine my future work self (我很容易想象出未来工作中的自己) 5. The mental picture of this future is very clear (我脑海里对这个未来的画面非常清晰)
**Growth mindset (Dweck et al., 1995)**
1. Everyone, no matter who they are, can significantly change their basic characteristics (无论是谁，每个人都可以显著地改变自己的基本特质) 2. No matter what kind of person someone is, they can always change very much (无论现在是什么样的人，人们总是可以有很大的改变) 3. All people can change even their most basic qualities (所有人都可以改变，即使是他们最基础的特质) 4. People can always substantially change the kind of person they are (人们总是可以极大地改变他们的特征特质) 5. As much as I hate to admit it, you can’t teach an old dog new tricks. People can’t really change their deepest attributes ^Reversed^ (虽然我不愿承认，但本性难移，人们无法真正改变他们最深层的特质) 6. Everyone is a certain kind of person, and there is not much that can be done to really change that ^Reversed^ (每个人都有其固有特质，很难真正去改变它) 7. The kind of person someone is, is something very basic about them and it can’t be changed very much ^Reversed^ (一个人的特质是他们最根本的东西，很难有大的改变) 8. People can do things differently, but the important parts of who they are can’t really be changed ^Reversed^ (人们可以有着不同的做事方式，但他们的核心特质很难改变)
**Knowledge hiding (Connelly et al., 2012)**
1. Agreed to help him/her but never really intended to (同意帮助他/她，但却从来没有打算真的帮忙) 2. Agreed to help him/her but instead gave him/her information different from what s/he wanted (同意帮助他/她，但提供的信息不是他/她想要的) 3. Told him/her that I would help him/her out later but stalled as much as possible (告诉他/她我稍后会帮助他/她，但是能拖一时是一时) 4. Offered him/her some other information instead of what he/she really wanted (给他/她提供一些其他信息，而不是他/她真的想要的信息) 5. Pretended that I did not know the information (假装我不知道那些信息) 6. Said that I did not know, even though I did (即使我知道那些信息，我也会告诉他/她我不知道) 7. Pretended I did not know what s/he was talking about (假装我不知道他/她在说什么) 8. Said that I was not very knowledgeable about the topic (告诉他/她我对这个话题也不是很了解) 9. Explained that I would like to tell him/her, but was not supposed to (解释说我想要告诉他/她，但我不应该这样做) 10. Explained that the information is confidential and only available to people on a particular project (解释说这一信息是机密信息，只有属于这个特定项目的人才能知道) 11. Told him/her that my boss would not let anyone share this knowledge (告诉他/她我的领导不希望让其他人知道这信息) 12. Said that I would not answer his/her questions (告诉他/她我不能回答他/她的问题)
